# PocketMaster provides a flexible and automated tool for analyzing, clustering, and visualizing structural diversity in protein pockets

**DOI:** 10.1038/s41598-026-47960-2

**Published:** 2026-06-22

**Authors:** Narek Abelyan

**Affiliations:** 1 Institute of Biomedicine and Pharmacy, Russian-Armenian University, Yerevan, Armenia; 2 Laboratory of Computational Modeling of Biological Processes, Institute of Molecular Biology, National Academy of Sciences of the Republic of Armenia (NAS RA), 0014 Yerevan, Armenia

**Keywords:** Protein pocket analysis, Structural clustering, RMSD calculation, Conformational diversity, Protein alignment, Structure-based drug design, Computational biology and bioinformatics, Drug discovery, Structural biology

## Abstract

PocketMaster is a flexible and automated tool for the analysis, clustering, and interpretation of protein pockets, enabling the exploration of structural diversity in functional and interacting regions of proteins. The tool provides multiple strategies for defining pocket alignment regions, along with various alignment algorithms and clustering approaches, allowing analyses to be customized for different research objectives. In addition, it automatically documents results and provides informative visualizations and reports. With these capabilities, PocketMaster can be particularly valuable in the early stages of drug design, where accurate analysis and selection of protein structures are essential. Using TYK2 as a case study, PocketMaster demonstrates its ability to identify conformational differences between kinase and pseudokinase domains, as well as subtypes of structures within each domain, reflecting the influence of various ligands and protein states. The estrogen receptor alpha (ERα) ligand-binding pocket was also analyzed as an additional case study, showing the tool’s performance in capturing conformational variations in helix 12 (H12) between active (agonist-bound) and inactive (antagonist-bound) states. The results confirm known structural features and illustrate the potential of the tool for systematic exploration of protein pockets, quantitative assessment of differences, and support of rational drug design. The PocketMaster source code, together with example input files and documentation, can be accessed at https://github.com/narek-abelyan/PocketMaster.

**Supplementary Information:**

The online version contains supplementary material available at https://doi.org/10.1038/s41598-026-47960-2.

## Introduction

The three-dimensional structure of a protein is not a static entity, but a dynamic system capable of adopting multiple conformational states^1^. Particularly important are the regions involved in the specific binding of small molecules or proteins - active sites, allosteric pockets, catalytic regions, and PPI pockets^2^. Even minor changes in these areas can significantly affect protein function. Conformational variations can be induced by various factors, including ligand binding, allosteric effects, changes in pH, temperature, or point mutations, especially if they occur near a functional site^3,4^.

Analyzing such changes is a key step in understanding the molecular mechanisms of protein function, as well as in selecting the optimal structure and conformation for drug development targeting specific protein states^5^. Comparing the pockets of the same protein under different conditions allows a deeper exploration of its structural diversity, revealing patterns that stabilize interactions or, conversely, uncovering mechanisms that disrupt function - for example, in the case of disease-related mutations. This can be particularly useful in the early stages of drug development, when precise analysis and selection of suitable protein structures are critical. Thousands of structures reflecting different functional states, mutant forms, and interactions with various ligands or inhibitors are already available in the PDB^6^. While experimental structures from the PDB offer direct insights into observed conformational states, computational frameworks can generate additional alternative conformations and model pocket flexibility beyond available crystal structures^7^. Despite the existence of numerous methods for protein structure comparison, such as ProBiS, PPS-align, FLAPP, G-LoSA, and PocketMatch 2.0, most of them are primarily focused on detecting structural similarities of pockets between different, often non-homologous proteins. These approaches are particularly useful for predicting binding sites, studying polypharmacology, and identifying new therapeutic targets. However, they are less suitable for detailed analysis of the conformational ensemble of a single protein, reflecting the effects of different ligands, activation states, mutations, and other factors - a task that is especially relevant in the early stages of structure-based drug design. Moreover, none of these methods represents a complete, convenient, and automated end-to-end workflow that integrates all key stages of analysis within a single system. In particular, they do not include methods for loading structures from various databases, preliminary preprocessing of protein structures, flexible pocket definition for subsequent alignment and similarity calculation, support for multiple alignment strategies, various clustering approaches, as well as visualization and report generation.

To address this issue, we developed PocketMaster, which fills this gap by providing a convenient, automated, and highly customizable end-to-end workflow. Implemented in Python and integrated with the PyMOL API^8^, PocketMaster provides a broad range of flexible settings, enabling analyses to be tailored to diverse research objectives and structural data types. The tool includes methods for loading structures from various databases and performing preliminary processing of protein structures. It also supports multiple approaches for defining pocket regions for subsequent alignment, a variety of RMSD calculation methods, and clustering algorithms, allowing researchers to select optimal parameters for each specific study. Additionally, PocketMaster automatically saves results and generates informative visualizations and reports. This comprehensive functionality ensures convenience, flexibility, and highly interpretable analysis of protein pockets, facilitating an in-depth examination of their structural features and patterns.

PocketMaster does not aim to replace existing tools, but rather to complement them by providing an integrated end-to-end workflow while also enabling the analysis of protein pocket conformational variability within a single protein or closely related protein groups by supporting more sensitive, fine-grained methods.

Beyond the conceptual differences mentioned above, the main algorithmic differences of PocketMaster compared to some other protein pocket comparison programs are summarized in Table 1.

**Table 1:** Comparison of protein pocket alignment methods and similarity metrics.

Name	Alignment type	Main similarity metrics
PocketMaster	Multiple alignment methods supported: align - Structure-based (sequence-assisted), cealign - Geometry-based structural alignment (sequence-independent), super - Structure-based (explicit residue mapping), rms, rms_cur - No alignment; RMSD calculation on already aligned structures	RMSD calculation over all atoms and Cα atoms (precise atomic-level metric)
ProBiS	Pocket Surface-patch matching / surface-based alignment (sequence-independent) Vertices are points in 3D space, and each replaces one functional group belonging to a residue on the protein surface. Each vertex is labeled with the physicochemical properties of a functional group that it replaces, hydrogen bond acceptor, hydrogen bond donor, mixed acceptor/donor, aromatic and aliphatic.	RMSD calculated only at the level of local surface patches (aligned surface patches)
PocketMatch	Structure alignment of protein pockets over Cα, Cβ, and side-chain centroids + residue chemistry (sequence-independent) Each pocket is represented as a set of points (Cα, Cβ, and side-chain centroids) labeled according to residue properties. Pairwise distances between points are computed and grouped by residue and point types, and alignment is performed by comparing these distance sets to find the largest number of mutually compatible distances (PMScore).	PMScore measures the similarity between two protein pockets as the fraction of matched pairwise distances between chemically labeled points.
FLAPP	Structure alignment of protein pockets over Cα + residue chemistry (sequence-independent) Each pocket is represented as a set of Cα atoms labeled according to residue properties (all 20 amino acids are grouped into five standard physicochemical classes), and alignment is performed by comparing distance matrices to find the largest set of mutually compatible pairs.	RMSD over Cα atoms after alignment.
G-LoSA	Local graph-based alignment on chemical feature points - CFP (sequence-independent) Each pocket is represented as a set of chemical feature points (CFPs) of amino acids, such as hydrogen-bond donors/acceptors, hydrophobic centers, aromatic rings, and other functional groups.	GA-score (ranging from 0 to 1) evaluates how well the chemical feature points match in terms of both distances and types.
PPS-align	Each pocket is represented as a set of Cα atoms from pocket residues (sequence-independent) Alignment is performed by greedily identifying a maximal set of geometrically compatible Cα–Cα pairs to obtain an optimal structural superposition and similarity score (PPS-score).	PPS-score, defined as the fraction of geometrically consistent Cα–Cα residue pairs between two pockets.

## Materials and methods

### Main functionalities of PocketMaster

PocketMaster offers a high degree of flexibility and configurability, allowing it to be adapted for a wide range of research tasks. The main customization features include:

1. Importing protein structures

PocketMaster supports several modes for obtaining three-dimensional protein structures:

- Local PDB files: Users can load files from a specified folder, which is convenient when working with proprietary or pre-processed data.
- UniProt ID: The software can automatically retrieve and load all available PDB structures corresponding to a given UniProt ID^9^.
- PDB ID: PocketMaster can identify the corresponding UniProt ID from a PDB ID and load all associated PDB structures.

2. Flexible preprocessing of structures

Before analysis, PocketMaster offers preprocessing, which can help standardize the data and ensure accurate structural comparisons. This includes removal of water molecules, ions (e.g., Mg²⁺, Cl⁻), sulfates, phosphates (SO₄, PO₄), buffer components (TRS, MES, HEPES, etc.), cryoprotectants (GOL, EDO, MPD, etc.), reducing agents (DTT, BME, TCEP, etc.), modified amino acid residues (CSO, MSE, SEP, TPO, PTR, etc.), alternate conformations (ALTLOC), hydrogen atoms, anisotropic parameters (ANISOU), and other elements not belonging to the main polymer chain.

3. Multiple approaches for defining alignment regions

**Fig. 1:**
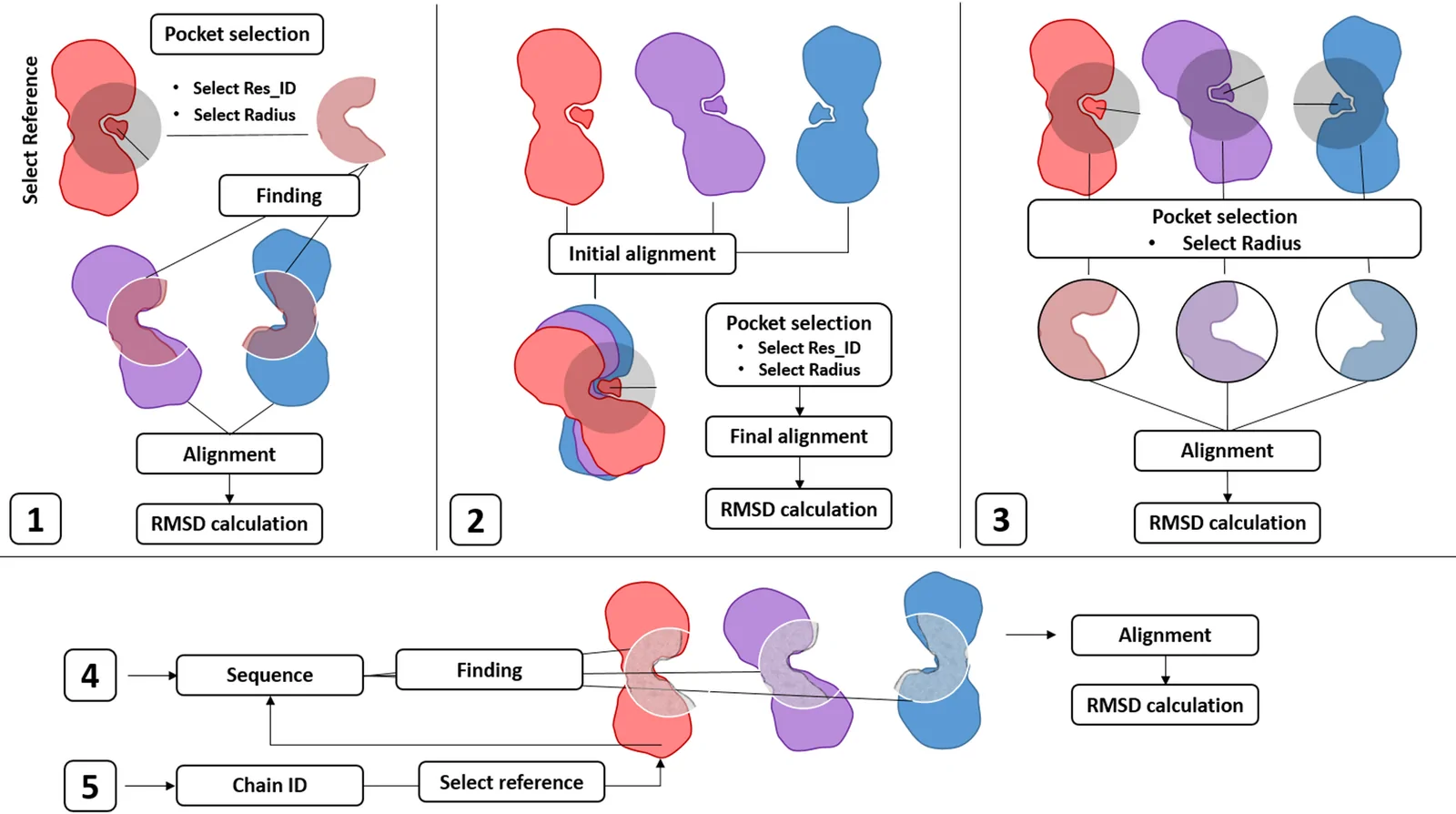
Five strategies implemented in PocketMaster for defining pocket regions used for subsequent alignment, with their detailed interpretation: 1 - The region is defined on the selected reference structure by a specified residue ID and radius (Å), and then this region is searched for and aligned in all structures. 2 - Prior to region definition, all structures are preliminarily aligned to the reference, after which the region is defined for each structure around the selected residue within a given radius (Å). 3 - The region is defined for each structure around its HET groups within a specified radius (Å). With proper preprocessing, it is possible to keep only ligands in the HET groups . 4 - The region is defined according to a user-provided list of residues, and then this region is searched for and aligned in all structures. 5 - The region is defined on the reference structure by a specified chain identifier, and then this region is searched for and aligned in all structures.

PocketMaster provides multiple methods for defining the alignment region and subsequent RMSD calculation, ensuring precise selection of functionally relevant areas. This feature is particularly important for analyzing complex or non-standard protein structures (Fig. 1).

1. The region is defined on the selected reference structure by a specified residue ID and radius (Å), and then this region is searched for and aligned in all structures.
2. The region is defined after a preliminary alignment of all structures to the reference, for each structure around the selected residue within a given radius (Å).
3. The region is defined for each structure around its HET groups within a specified radius (Å). With proper preprocessing, it is possible to keep only ligands in the HET groups.
4. The region is defined according to a user-provided list of residues, and then this region is searched for and aligned in all structures.
5. The region is defined on the reference structure by a specified chain identifier, and then this region is searched for and aligned in all structures.

4. Alignment and RMSD calculation methods

PocketMaster supports multiple algorithms for structural alignment and RMSD calculation, each suitable for different analysis scenarios. Users can select the most appropriate method depending on the task, required accuracy, the presence of unmatched atoms between structures, and computational efficiency:

1. align – A strict alignment method that matches residues according to the amino acid sequence and performs an optimal superposition of the moving structure onto the target. Atoms without a corresponding match between structures are excluded from the calculation. RMSD is then computed based on the matched atoms. Atoms without correspondence or those deviating strongly (beyond the default cutoff of 2 Å) may be excluded from the RMSD calculation. This method is suitable for high-accuracy similarity assessments of structures with similar sequences.
2. cealign – A structural alignment method based on the Combinatorial Extension (CE) algorithm. It aligns protein structures by the geometry of their fragments, minimizing the RMSD between matching Cα atoms. In the final stage of the global alignment, RMSD is calculated only for the Cα atoms of matched segments, while other atoms and non-matching residues are ignored in the calculations, although they move together with the moving structure. This method is particularly effective for comparing structures with different sequences, varying conformations, or mismatched individual atoms.
3. super – A more flexible algorithm, a modified alignment method similar to align, but more robust to sequence mismatches and residue numbering differences. It performs structural matching while accounting for insertions, deletions, and partial mismatches, providing more reliable alignments when there are moderate differences between structures.
4. rms – A direct RMSD calculation between two structures without alignment, assuming a complete one-to-one correspondence of atoms in order. This method is sensitive to mismatches and does not allow differences in structural composition. It is used to assess identical or strictly fitted structures when preliminary alignment has already been performed.
5. rms_cur – A simplified and faster method similar to rms, but with lower computational overhead. It assumes complete atom correspondence and a fixed sequence. This method is suitable for screening large numbers of structures when a quick, though less precise, assessment is required.

Each method is implemented using PyMOL’s built-in tools, ensuring reproducibility of results and compatibility with standard practices in structural analysis.

5. RMSD calculation over all atoms or only Cα atoms

Users can choose to compute RMSD using either all atoms or only α-carbon atoms, allowing flexibility in the accuracy and sensitivity of similarity assessments depending on the specific task and the quality of the data.

6. Hierarchical clustering methods

PocketMaster implements several agglomerative hierarchical clustering algorithms, allowing protein sites to be grouped based on their structural similarity (expressed as an RMSD distance matrix). Users can select the most suitable method depending on the nature of the data and the research objectives. All algorithms are implemented using the SciPy library^10^ and rely on a standard distance metric (typically RMSD calculated after alignment):

1. ward – Merges clusters in a way that minimizes differences within each group. It works best with Euclidean distances (e.g., RMSD) and produces compact clusters that are balanced in size. This method is particularly useful when it is important for structures within the same cluster to be as similar as possible.
2. single – Uses the minimum distance between elements of different clusters (also known as the nearest-neighbor method). It can lead to elongated clusters with a linear structure and is highly sensitive to outliers. Particularly useful for sequentially merging similar structures when tracking gradual changes is important.
3. complete – Merges clusters based on the largest distance between their elements (farthest-neighbor method). This results in more compact and clearly separated groups. This method is suitable when it is important to reliably separate different clusters. It is also less sensitive to outliers but can sometimes merge fairly dissimilar structures prematurely.
4. average (UPGMA) – Calculates the distance between clusters as the average of all pairwise distances between elements from the two clusters. Positioned between the single method (minimum distance) and the complete method (maximum distance), it provides a more balanced and even division of groups. This approach is often used for constructing evolutionary and structural trees.
5. centroid – Measures the distance between clusters based on their centroids, i.e., the mean positions of all points in each cluster. This approach usually gives clear results but can sometimes produce incorrect clustering if non-standard distance measures are used, potentially resulting in an unordered cluster tree.
6. median – Similar to centroid but uses the median instead of the mean. More robust to outliers, though less commonly used in bioinformatics.
7. weighted (WPGMA) – A simplified variant of the average method, where the “weight” (importance) of clusters does not change during merging. This method is used less frequently but can be useful when the data has a known hierarchical structure and it is important to preserve the influence of the original groups without distortion during merging.

All methods are compatible with customizable clustering stop criteria (by number of clusters, distance threshold, or automatic estimation), making the analysis highly flexible.

7. Clustering parameters:

PocketMaster provides three main options for forming clusters:

1. By number of clusters (maxclust) - The user specifies the desired number of clusters in advance. The algorithm merges elements until the specified number is reached. This approach is useful when the approximate structure of the data is known or when a specific number of groups is required for further analysis.
2. By distance threshold (distance) - Clusters are formed until the distances between groups exceed a specified threshold. This allows control over intra-cluster similarity: the lower the threshold, the more homogeneous the groups. This method is particularly suitable when clearly defined boundaries between clusters are important.
3. Automatic mode - The algorithm determines the optimal number of clusters based on the data and predefined quality criteria. This mode is useful when the number of clusters is unknown or when the goal is to obtain the most informative grouping without manual tuning. Two approaches are available for automatic clustering:

- Elbow method - The number of clusters is selected at the “elbow” point of a plot showing within-cluster distance versus number of merges, where additional clusters result in minimal improvement in compactness.
- Threshold based on 70% of the maximum merge distance - Clusters are formed until the distance between merging groups exceeds 70% of the maximum observed distance, allowing identification of naturally homogeneous groups. It is a practical heuristic, used by default in SciPy/Seaborn functions. This threshold allows large branches to be clearly separated, making the dendrogram more readable.

The ability to choose the clustering method provides greater flexibility and allows the analysis to be adapted to specific tasks, improving the interpretation of results and the quality of the identified groups.

8. Saving preprocessed, aligned structures and analysis results

Saving allows easy storage, viewing, and reuse of data, simplifying the integration of PocketMaster into research workflows and ensuring reproducibility.

9. Generating concise reports on structural and sequence differences of pockets

The reports provide key information on variations in the amino acid composition of selected structural regions, facilitating their comparison and interpretation.

10. Creating clear visualizations: heatmaps, dendrograms, and histograms

Visualizations help identify recurring patterns, group similar structures, and quickly assess overall similarities and differences.

In summary, PocketMaster offers researchers a wide range of configurable options, enabling accurate and comprehensive analysis of protein sites, while allowing the workflow to be adapted to specific scientific tasks and data volumes.

### Practical considerations for predicted structures

When working with predicted protein structures, such as those generated by AlphaFold, special attention should be paid to the confidence of structural regions. AlphaFold provides per-residue confidence scores (pLDDT), which can be used to assess the reliability of local structural features. Low-confidence regions, typically corresponding to flexible loops or disordered segments, may introduce significant noise into pocket definition, structural alignment, and RMSD calculations. Therefore, we recommend filtering or masking residues with low pLDDT scores (e.g., below 70), depending on the desired level of stringency. In practical applications, excluding such regions can improve the robustness of pocket comparison and lead to more biologically meaningful clustering results.

### Program launch modes and settings

PocketMaster offers two main modes of operation. In the interactive mode, users enter analysis parameters manually via the console, allowing flexible real-time adjustment of the task. To run the program in this mode, the following command is used:

- python PocketMaster.py

The second mode uses a YAML configuration file, where all necessary parameters are predefined. This ensures automation and reproducibility of the analysis, especially when working with large datasets or when repeated runs with identical settings are required. The program is launched with a configuration file using the command:

- python PocketMaster.py --config config.yaml

When running in interactive mode, a run_config.txt file is generated at the end. It automatically saves all parameters entered by the user, allowing you to run the script later in automatic mode with the same settings, without re-entering parameters. This approach enhances the convenience and efficiency of using PocketMaster in various research scenarios.

## Results

### Application of PocketMaster on TYK2: analysis of ligand-binding pocket variability

To demonstrate the capabilities and practical application of PocketMaster, we performed a structural analysis of the TYK2 protein (Tyrosine kinase 2) - a member of the Janus kinase family that plays a crucial role in cytokine signaling and immune regulation^11–13^. TYK2 is considered a promising therapeutic target for autoimmune disorders such as psoriasis, rheumatoid arthritis, and systemic lupus erythematosus^11,14,15^. In recent years, efforts to develop selective TYK2 inhibitors have resulted in a large number of crystal structures of the protein in complex with various ligands^13,16–18^, making TYK2 a good subject for analyzing the conformational diversity of its ligand-binding pocket.

The starting dataset was built using UniProt ID: P29597, from which all available TYK2 crystal structures were automatically retrieved from the Protein Data Bank (PDB). The dataset includes structures representing different TYK2 domains. We excluded non-TYK2 or incomplete structures: 8EXN and 8EYC are PTP1B co-crystals containing TYK2 peptides, and 4PO6 contains only the FERM and SH2 domains (residues 26–566) and lacks both the pseudokinase and kinase domains. Users should therefore verify that the input structures contain the target domain or pocket, as this is not automatically validated by the tool. In total, 49 structures were collected, differing in bound ligand type, activation state, and, in some cases, the presence of point mutations.

To standardize the input data, preprocessing was performed (do_preprocess = 1), which included the removal of water molecules (clean_options:1 - water), ions (clean_options:2 - MG, CL, etc.), sulfates and phosphates (clean_options:3 - SO4, PO4, etc.), buffer components (clean_options:4 - TRS, MES, etc.), cryoprotectants (clean_options:5 - GOL, EDO, etc.), reducing agents (clean_options:6 - DTT, BME, TCEP), alternative atom conformations (clean_options:10 - altloc), and anisotropic parameters (clean_options:11 - anisou). This step removed structural artifacts and improved the quality of subsequent alignments.

All structures were preliminarily aligned to a reference structure (ref_align = 6NZP, init_align = 1, init_align_chain_id = “A”). The reference structure chosen for alignment was PDB ID: 6NZP, containing the inhibitor LB7 tightly bound in the active site. The binding pocket was defined individually for each structure (pocket_method:2) around the reference ligand LB7 (ligand_resi: 901, ligand_chain: A) using a 7 Å radius (radius:7), covering the entire ligand-binding region and including all residues potentially involved in ligand recognition and stabilization.

To evaluate the structural similarity of binding pockets across all TYK2 structures, an all-vs-all comparison was performed (comparison_mode:1). The RMSD values were calculated using the super algorithm (rmsd_method:2 - super), which allows flexible alignment even in the presence of partial atom mismatches between compared structures. The procedure generated a complete RMSD distance matrix representing the pairwise pocket dissimilarities among all structures.

Subsequently, hierarchical clustering was applied using the average linkage (UPGMA) method (linkage_method:4 - average (UPGMA)), while the optimal clustering threshold was automatically determined via the elbow method (cl_choice:3 - Elbow method).

The PocketMaster parameters used in this case study (e.g., pocket radius, alignment method, clustering linkage, and automatic clustering heuristics) are provided as reasonable defaults rather than fixed or optimized choices, facilitating reproducible analyses and initial exploration. PocketMaster is explicitly designed to encourage exploration of alternative settings. The goal of the manuscript is to demonstrate the workflow and capabilities of the tool rather than prescribe a single “best” set of parameters for all applications. Users can easily modify these settings depending on the system and research question, and alternative choices may lead to different cluster boundaries.

**Fig. 2:**
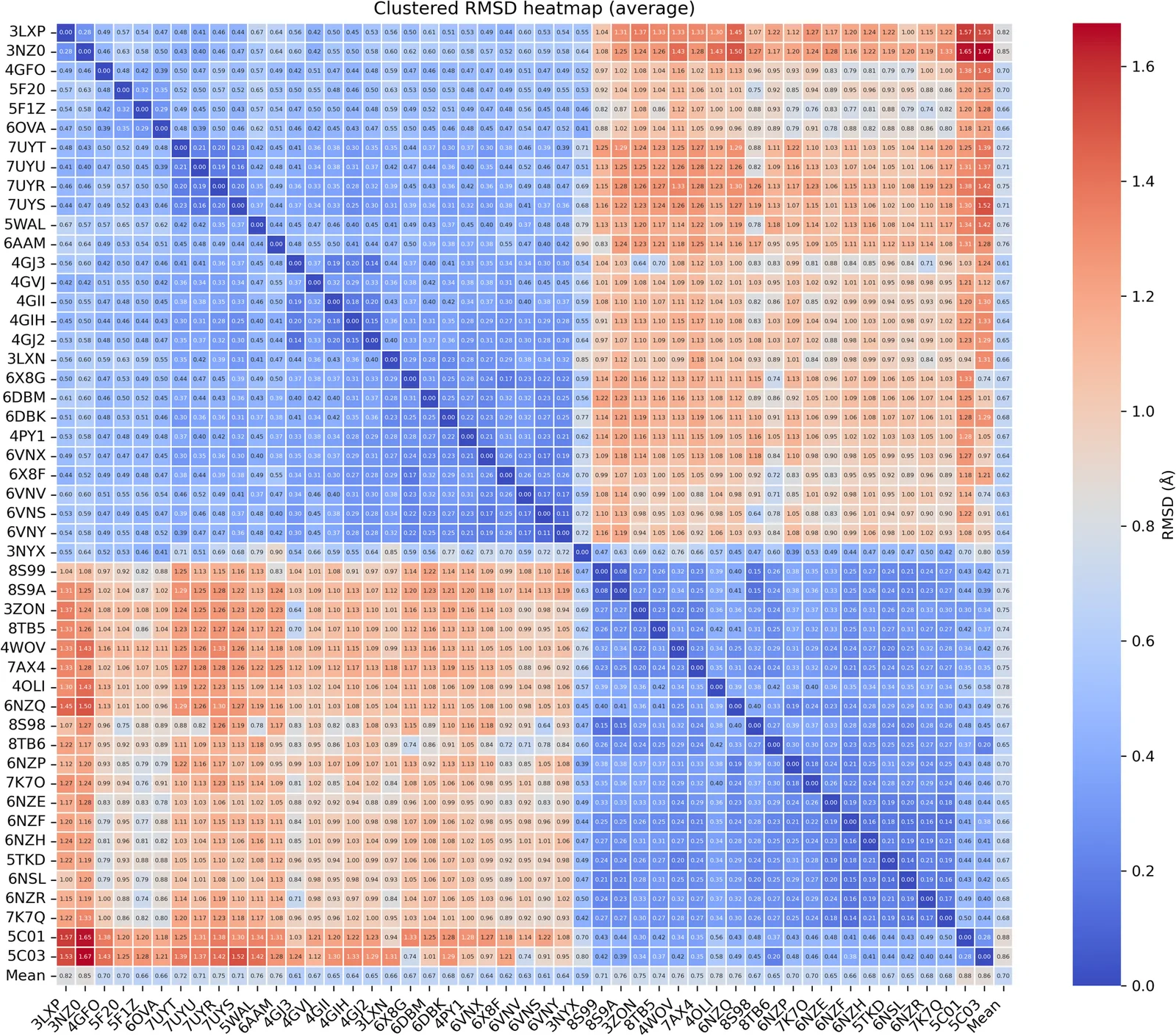
Ordered Heatmap of the pairwise RMSD matrix for TYK2 structures. Each cell in the heatmap represents the RMSD value between the binding pockets of two TYK2 structures. The block-diagonal pattern highlights the separation of structures into two main domains: the kinase domain (upper-left block) and the pseudokinase domain (lower-right block). Cells within a block correspond to RMSD comparisons within the same domain, showing low structural variation (blue), while off-diagonal blocks correspond to between-domain comparisons, showing higher RMSD values(red/orange). This organization clearly visualizes the conformational differences between domains and the relative similarity among structures within each domain.

**Fig. 3:**
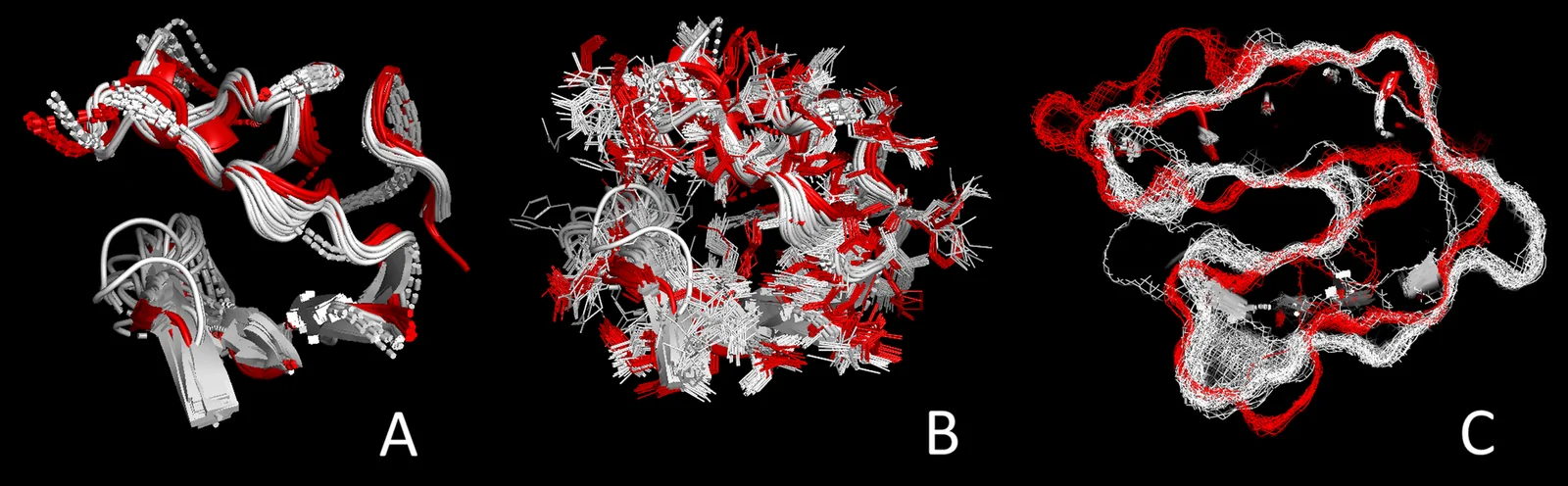
Three-dimensional alignment and structural comparison of TYK2 binding pockets from the kinase and pseudokinase domains. Structures containing the kinase pocket are shown in red, whereas structures with the pseudokinase pocket are shown in white. The binding pockets are visualized using different representations: (**A**) cartoon, (**B**) cartoon with side chains shown as lines, and (C) surface mesh representation.

This workflow enabled the identification of distinct groups of models sharing high conformational similarity. Clustering results were visualized as distance distribution histograms, an RMSD heatmap, and a dendrogram. According to the results, all TYK2 structures were clearly classified into two main classes, which differ significantly in conformation (Fig. 2, blue and red clusters on the heatmap; Fig. 3). These pronounced structural differences correspond to the well-characterized kinase and pseudokinase domains, which differ substantially in their spatial organization^13,17,19^. This correct classification does not represent a novel finding but serves as a validation of PocketMaster’s methodology, demonstrating its ability to accurately capture structural differences and reliably cluster protein pockets. This validation therefore supports the use of the tool for systematic exploration of structural diversity and quantitative assessment of differences.

Structures with PDB IDs: 3NYX, 3LXP, 3NZ0, 7UYS, 6DBK, 6VNX, 6 × 8G, 4GFO, 6OVA, 5F1Z, 5F20, 6DBM, 6 × 8 F, 4PY1, 6VNV, 6VNS, 6VNY, 4GVJ, 4GIH, 4GII, 4GJ2, 4GJ3, 3LXN, 7UYR, 7UYT, 7UYU, 5WAL and 6AAM represent kinase domains.

Structures with PDB IDs: 5C01, 5C03, 4OLI, 8S98, 8S99, 8S9A, 4WOV, 8TB6, 3ZON, 7AX4, 8TB5, 6NZE, 7K7Q, 6NZR, 5TKD, 6NSL, 6NZF, 6NZH, 7K7O, 6NZP, and 6NZQ contain the pseudokinase domain.

**Fig. 4:**
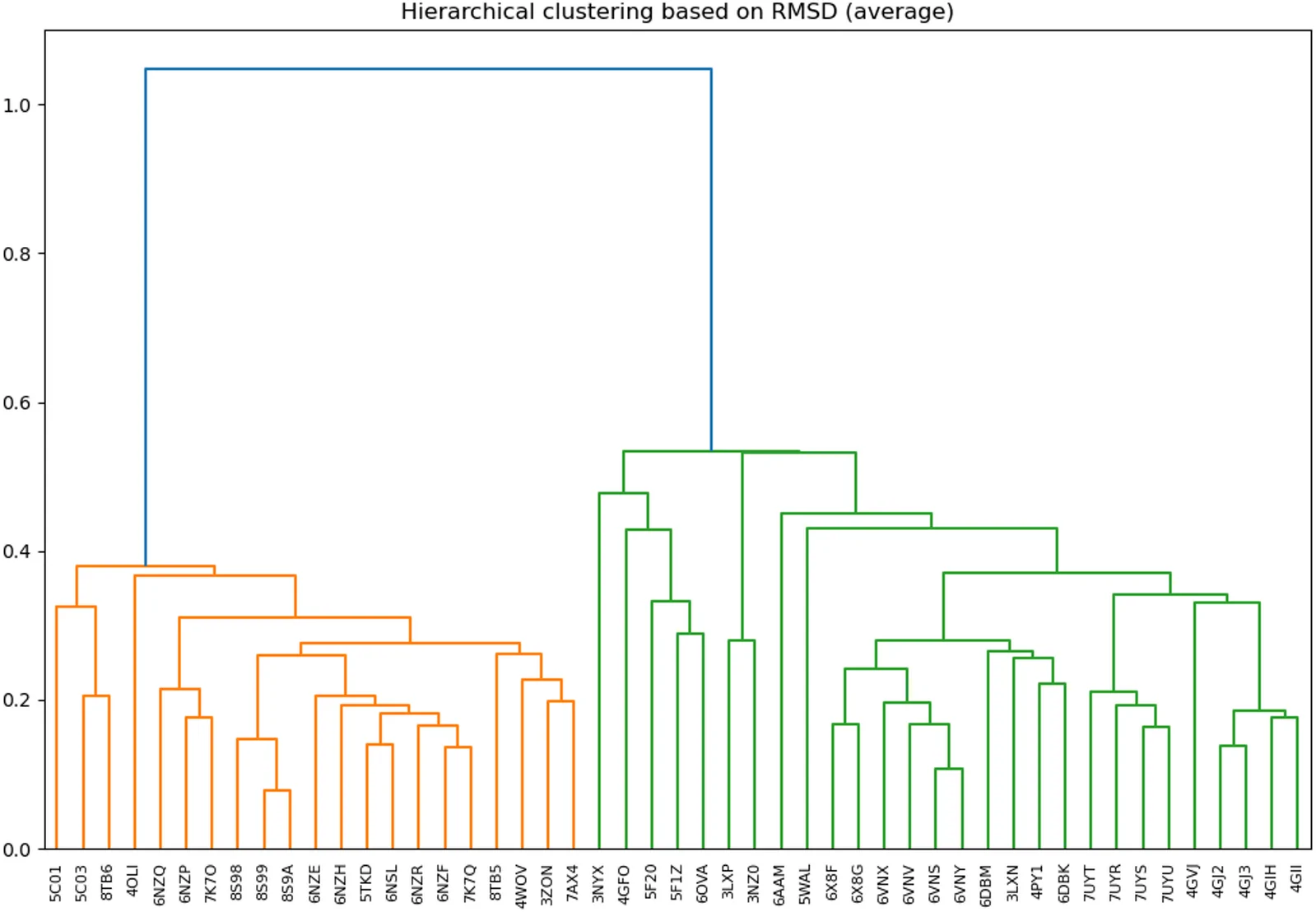
Hierarchical clustering dendrogram of TYK2 binding pockets. The dendrogram was generated using the average linkage (UPGMA) method based on the pairwise RMSD distance matrix. The clustering threshold was determined automatically using the elbow method. Each leaf represents a single TYK2 structure, and branch lengths correspond to RMSD values, indicating the degree of structural dissimilarity between clusters or individual structures. This visualization highlights the separation between kinase (green) and pseudokinase (orange) domains, as well as subgroups within each domain influenced by ligand type and protein conformation.

**Fig. 5:**
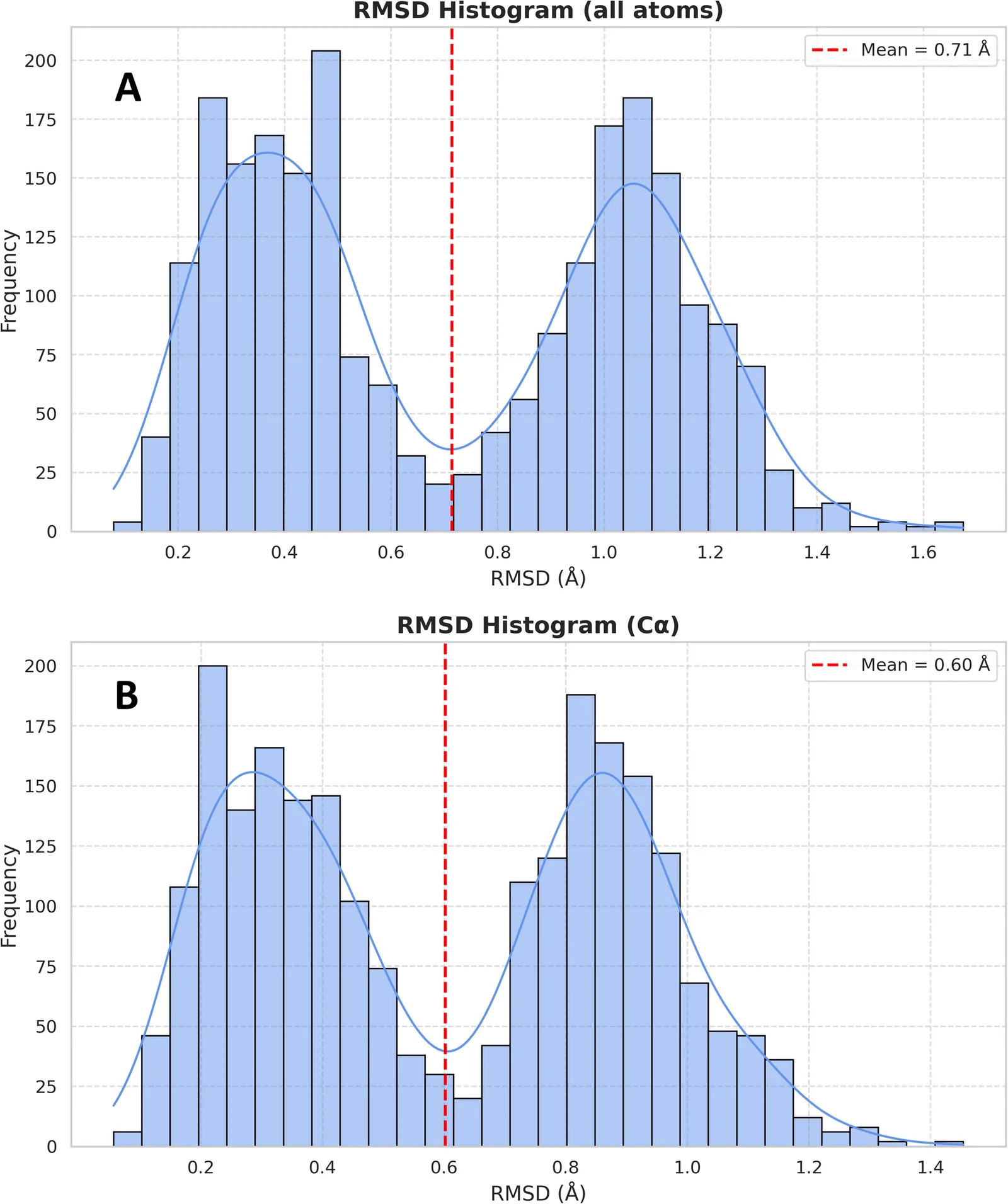
RMSD distributions for TYK2 binding pockets. Histograms show the distribution of RMSD values calculated over all atoms (**A**) and Cα atoms only (**B**). The bimodal shape reflects two types of comparisons: within-domain RMSD values (first peak, lower RMSD, structures from the same domain) and between-domain RMSD values (second peak, higher RMSD, comparisons between kinase and pseudokinase domains). The red dashed line indicates the mean RMSD.

Among these two domains, the pseudokinase domain binds deucravacitinib, a recently developed first-in-class allosteric TYK2 inhibitor^20,21^. The RMSD histograms and hierarchical clustering dendrogram further support this conclusion (Figs. 4 and 5), showing a clear separation of structures into two distinct clusters and highlighting the fundamental structural differences between the kinase and pseudokinase domains of TYK2.

**Fig. 6:**
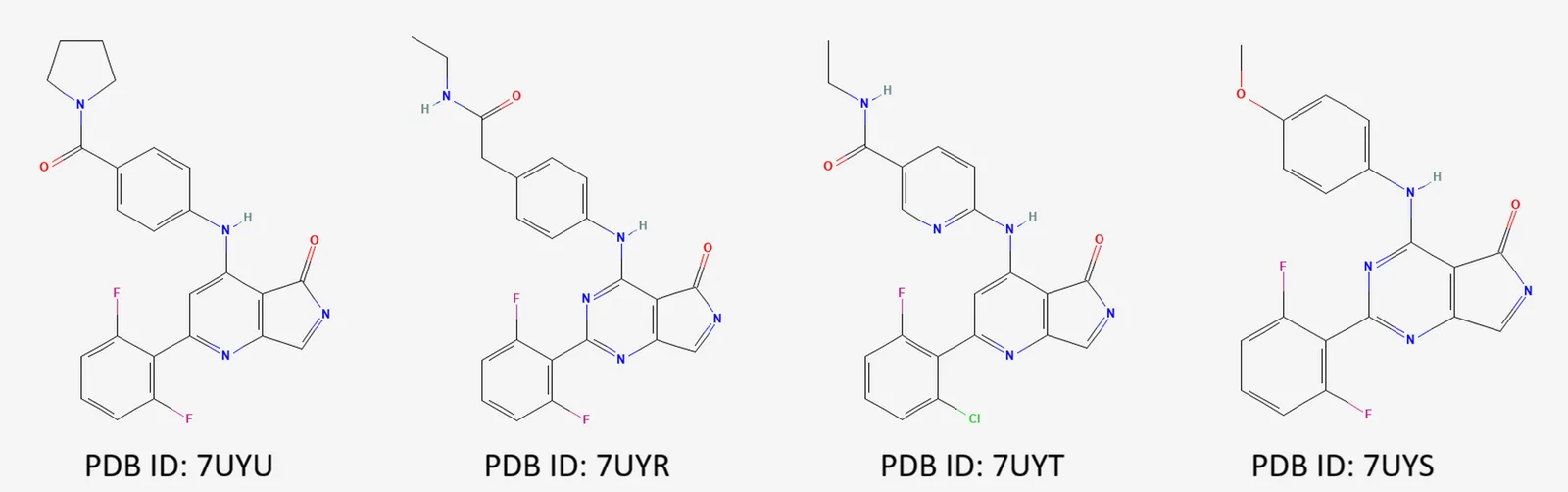
Comparison of ligands from PDB structures 7UYU, 7UYR, 7UYT, and 7UYS.

Further analysis revealed that within each of these classes, there are subgroups of structures that are either closely related or noticeably different from one another. These differences are influenced both by the type of bound ligand and by specific structural variations in the protein. Some structures formed dense, homogeneous clusters, while others exhibited pronounced deviations, potentially indicating unique conformations or ligand-induced rearrangements. For example, structures with PDB IDs 7UYU, 7UYR, 7UYT, and 7UYS contain highly similar ligands sharing a common chemical scaffold (Fig. 6), which explains their proximity and clustering within a single group.

These compounds share a common chemical scaffold originating from the same drug discovery program. Structural similarity among these ligands leads to similar protein-ligand interactions, reflected in the RMSD-based clustering of the pocket residues.

### Application of PocketMaster on estrogen receptor alpha binding pocket

**Fig. 7:**
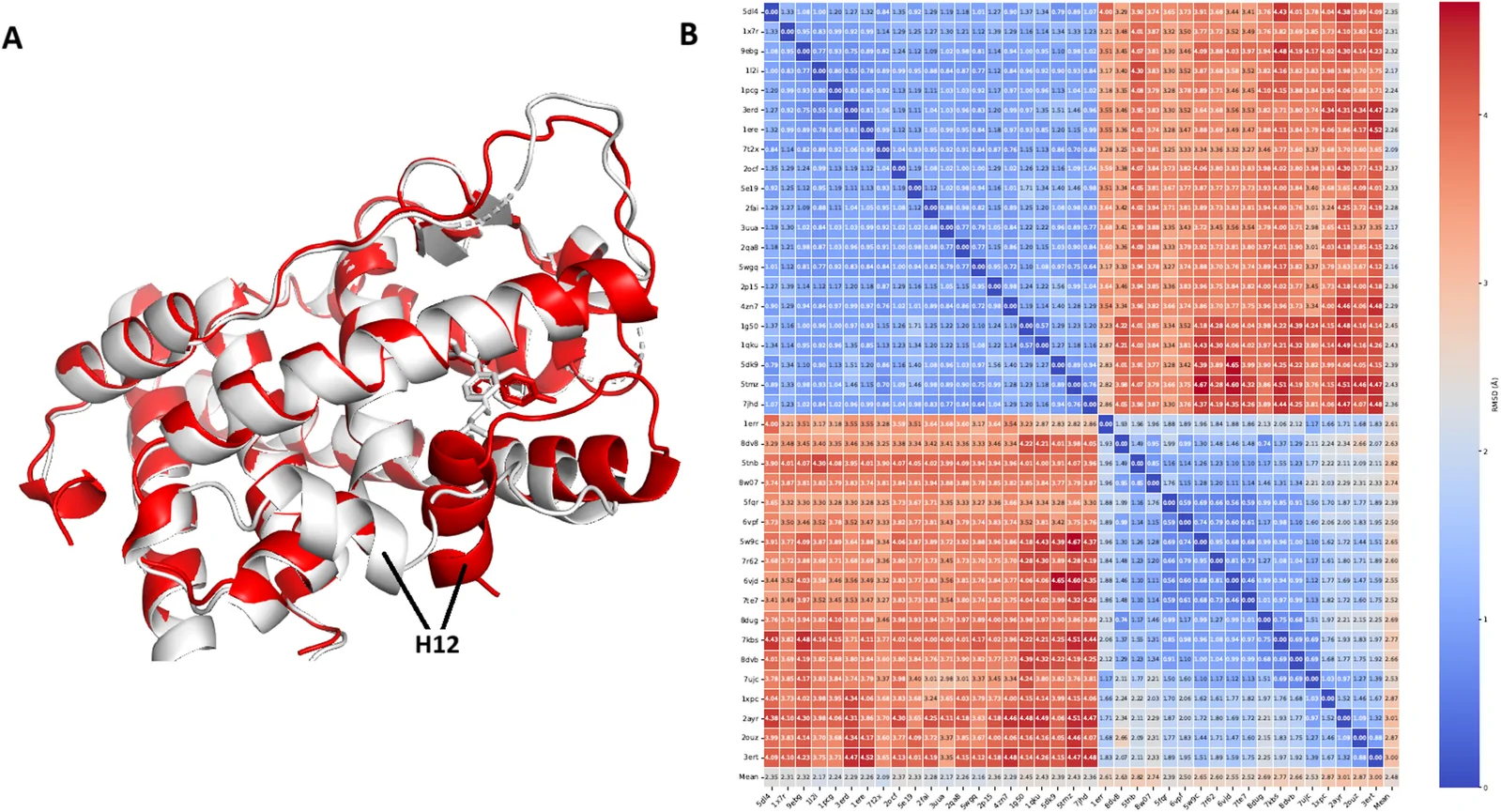
Conformational variability of the ligand-binding pocket of estrogen receptor α (ERα). (**A**) Structural illustration of the ERα ligand-binding domain highlighting the position of helix 12 (H12), a key structural element that controls receptor activity. H12 can adopt two alternative conformations corresponding to active (red) and inactive (white) receptor states. (**B**) RMSD heatmap of ERα ligand-binding pockets calculated using PocketMaster.

To further demonstrate the applicability of PocketMaster, we analyzed the ligand-binding pocket of estrogen receptor alpha (ERα), a nuclear receptor whose activity is strongly influenced by the position of helix 12 (H12). This helix can adopt two major conformations: an active position, typically associated with agonist-bound structures, and an inactive position, commonly observed in complexes with antagonists (Fig. 7A). A local dataset containing multiple ERα crystal structures representing both conformational states of H12 was collected (PDB IDs: 8W07, 8DVB, 8DV8, 8DUG, 7UJC, 7TE7, 7R62, 7KBS, 6VPF, 6VJD, 5W9C, 5TNB, 5FQR, 3ERT, 2OUZ, 2AYR, 1XPC, 1ERR, 9EBG, 7T2X, 7JHD, 5WGQ, 5TMZ, 5E19, 5DL4, 5DK9, 4ZN7, 3UUA, 3ERD, 2QA8, 2P15, 2OCF, 2FAI, 1 × 7R, 1QKU, 1PCG, 1L2I, 1G50, 1ERE). All structures were aligned and analyzed using PocketMaster with standard parameters. The only modification was the pocket radius, which was increased to 15 Å around the ligand to ensure that the H12 region contributing to pocket formation was included in the analysis. As a result, the program clearly separated and clustered the pocket structures into two distinct groups corresponding to the different conformations of H12 (Fig. 7B). This result further demonstrates the ability of PocketMaster to detect functionally relevant conformational differences in protein binding pockets (Supplementary Materials).

### Limitations

- The tool requires prior knowledge of the pocket location and does not identify pockets *de novo*.
- Automatic selection of the number of clusters may not be optimal for all datasets.
- Computational benchmarks for scaling behavior are not provided.
- RMSD-based comparisons have known limitations for highly flexible binding sites.

## Conclusion

An important aspect of protein structural analysis is the concept of druggability, which refers to the ability of a protein to bind drug-like molecules with high affinity and specificity. Protein binding pockets are inherently dynamic and often exist in multiple conformational states, which can significantly influence ligand recognition and druggability [^22, 23^ ]. Many binding sites fluctuate between open, partially occluded, or transient conformations, and these structural variations can be critical for understanding how small molecules interact with the protein. Additionally, many proteins exhibit dynamic conformational behavior, where potentially druggable pockets may only appear transiently or under specific structural states. In this context, PocketMaster provides a valuable tool for exploring conformational diversity through clustering of pocket geometries across multiple structures. By grouping similar pocket conformations, the method can help identify recurring or rare structural states that expose transient binding sites. This is particularly relevant for proteins that are traditionally considered difficult to target, where static structures may fail to reveal cryptic or inducible pockets. Thus, PocketMaster can assist in uncovering new opportunities for structure-based drug design.

PocketMaster provides a user-friendly, automated, and fully customizable end-to-end workflow for protein pocket structural analysis. Its flexible design integrates diverse pocket definition strategies, alignment methods, RMSD calculation options, and clustering algorithms, enabling broad applicability across various research contexts.

Application of PocketMaster to the analysis of TYK2 ligand-binding pocket variability revealed a distinct structural separation into two domains - kinase and pseudokinase - as well as conformational subtypes within these domains associated with ligand type and protein states. Similarly, analysis of the estrogen receptor alpha (ERα) ligand-binding pocket captured functionally relevant conformational differences in helix 12 (H12), clearly distinguishing active (agonist-bound) and inactive (antagonist-bound) states. These findings not only confirm previously established structural characteristics of TYK2 and ERα but also underscore the potential of PocketMaster for data systematization and quantitative assessment of structural diversity, contributing to a deeper understanding of functionally relevant regions of proteins and supporting the selection of representative structures for drug development. Thus, PocketMaster can serve as a powerful tool in the arsenal of structural bioinformaticians and drug designers, enabling deeper understanding of protein structural diversity, identification of stable conformations, and selection of promising target forms for rational drug design.

## Supplementary Information

Below is the link to the electronic supplementary material.

Supplementary Material 1

## Data Availability

All data and software used in this study are freely available. The PocketMaster source code, together with example input files and documentation, can be accessed at https://github.com/narek-abelyan/PocketMaster.

## References

[1] Stank, A., Kokh, D. B., Fuller, J. C. & Wade, R. C. Protein binding pocket dynamics. *Acc. Chem. Res.* **49** (5), 809–815 (2016). 27110726 10.1021/acs.accounts.5b00516

[2] Seo, M. H., Park, J., Kim, E., Hohng, S. & Kim, H. S. Protein conformational dynamics dictate the binding affinity for a ligand. *Nat. Commun.* **5** (1), 3724 (2014). 24758940 10.1038/ncomms4724

[3] Chu, W. T. & Zheng, Q. C. Conformational changes of enzymes and DNA in molecular dynamics: Influenced by pH, temperature, and ligand. *Adv. Protein Chem. Struct. Biol.* **92**, 179–217 (2013). 23954102 10.1016/B978-0-12-411636-8.00005-5

[4] Wankowicz, S. A., Robinson, R. M., Kritikos, G., Hekstra, D. R. & Fraser, J. S. Ligand binding remodels protein side-chain conformational heterogeneity. *eLife* **11**, e74114 (2022). 10.7554/eLife.74114 PMC9084896 35312477

[5] Meller, A., de Groot, B. L. & Grubmüller, H. Toward physics-based precision medicine: Exploiting protein dynamics to design new therapeutics and interpret variants. *Protein Sci.* **33** (3), e4902 (2024). 10.1002/pro.4902 PMC10868452 38358129

[6] Burley, S. K. et al. The single global macromolecular structure archive. In *Protein Crystallography: Methods and Protocols* 627–641 (Humana, 2017). 10.1007/978-1-4939-7000-1_26 PMC5823500 28573592

[7] Ding, W. et al. SAMF: A self-adaptive protein modeling framework. *Bioinformatics* **37** (22), 4075–4082 (2021). 34042965 10.1093/bioinformatics/btab411

[8] DeLano, W. L. PyMOL: An open-source molecular graphics tool. CCP4 Newsl. *Protein Crystallogr.* **40**, 82–92 (2002).

[9] UniProt Consortium. UniProt: A Worldwide Hub of Protein Knowledge. *Nucleic Acids Res.* **47** (D1), D506–D515 (2019). 30395287 10.1093/nar/gky1049 PMC6323992

[10] Gommers, R. et al. SciPy 1.15.0. Zenodo. (2024).

[11] Kang, C. et al. TYK2 targeting in immune-mediated inflammatory diseases. *Int. J. Mol. Sci.* **24** (4), 3391 (2023). 36834806 10.3390/ijms24043391 PMC9959504

[12] O’Shea, J. J., Gadina, M., Siegel, R., Farber, J. M. & Darnell, J. E. The JAK–STAT pathway: Impact on human disease and therapeutic intervention. *Nat. Rev. Drug Discov*. **14**, 75–92 (2015). 25587654 10.1146/annurev-med-051113-024537 PMC5634336

[13] Lupardus, P. J. et al. Structure of the pseudokinase–kinase domains from TYK2 reveals a mechanism for JAK autoinhibition. *Proc. Natl. Acad. Sci. U.S.A*. **111** (22), 8025–8030 (2014). 10.1073/pnas.1401180111 PMC4050602 24843152

[14] Moslin, R. et al. Highly selective inhibition of tyrosine kinase 2 (TYK2) for the treatment of autoimmune diseases: Discovery of the allosteric inhibitor BMS-986165. *J. Med. Chem.* **62** (7), 3893–3912 (2019). 10.1021/acs.jmedchem.9b00444 31318208

[15] Jensen, M. A. et al. Allosteric TYK2 inhibition: Redefining autoimmune disease therapy beyond JAK1–3 inhibitors. *Cell. Mol. Life Sci.* **80**, 163 (2023). 37863021 10.1016/j.ebiom.2023.104840 PMC10589750

[16] Chen, X. J. et al. A novel highly selective allosteric inhibitor of TYK2 can block inflammation- and autoimmune-related pathways. *Cell. Commun. Signal.* **21**, 287 (2023). 37845748 10.1186/s12964-023-01299-7 PMC10578023

[17] Min, X. et al. Structural and functional characterization of the JH2 pseudokinase domain of TYK2. *J. Biol. Chem.* **290** (45), 27261–27274 (2015). 26359499 10.1074/jbc.M115.672048 PMC4646372

[18] Argiriadi, M. A. et al. Enabling structure-based drug design of TYK2 through co-crystallography with a stabilizing ligand. *BMC Struct. Biol.* **12**, 22 (2012). 22995073 10.1186/1472-6807-12-22 PMC3478977

[19] Garrido-Trigo, A. et al. Molecular structure and function of janus kinases. *ECCO J* (2020). 10.1093/ecco-jcc/jjz206 PMC7395311 32083640

[20] Roskoski, R. Jr. Deucravacitinib is an allosteric TYK2 protein kinase inhibitor. *Pharmacol. Res.* **188**, 106651 (2023). 10.1016/j.phrs.2022.106642 36754102

[21] Kalola, U. K. *Deucravacitinib* (StatPearls Publishing, 2024). 39163466

[22] Al-Sanea, M. M. et al. Identification of non-classical hCA XII inhibitors using combination of computational approaches for drug design and discovery. *Sci. Rep.* **11**, 15516 (2021). 34330958 10.1038/s41598-021-94809-x PMC8324906

[23] Matevosyan, M. et al. Design of new chemical entities targeting both native and H275Y mutant influenza A virus by deep reinforcement learning. *J. Biomol. Struct. Dyn.* **41**, 10798–10812 (2023). 10.1080/07391102.2022.2158936 36541127

