# Supplementary material for: PocketMaster provides a flexible and automated tool for analyzing, clustering, and visualizing structural diversity in protein pockets
Source: Sci Rep. 2026 Jun 22;16:28695. doi: 10.1038/s41598-026-47960-2 (PMC13575124; doi:10.1038/s41598-026-47960-2)
Supplement: Supplementary file 1 [file 41598_2026_47960_MOESM1_ESM.pdf]

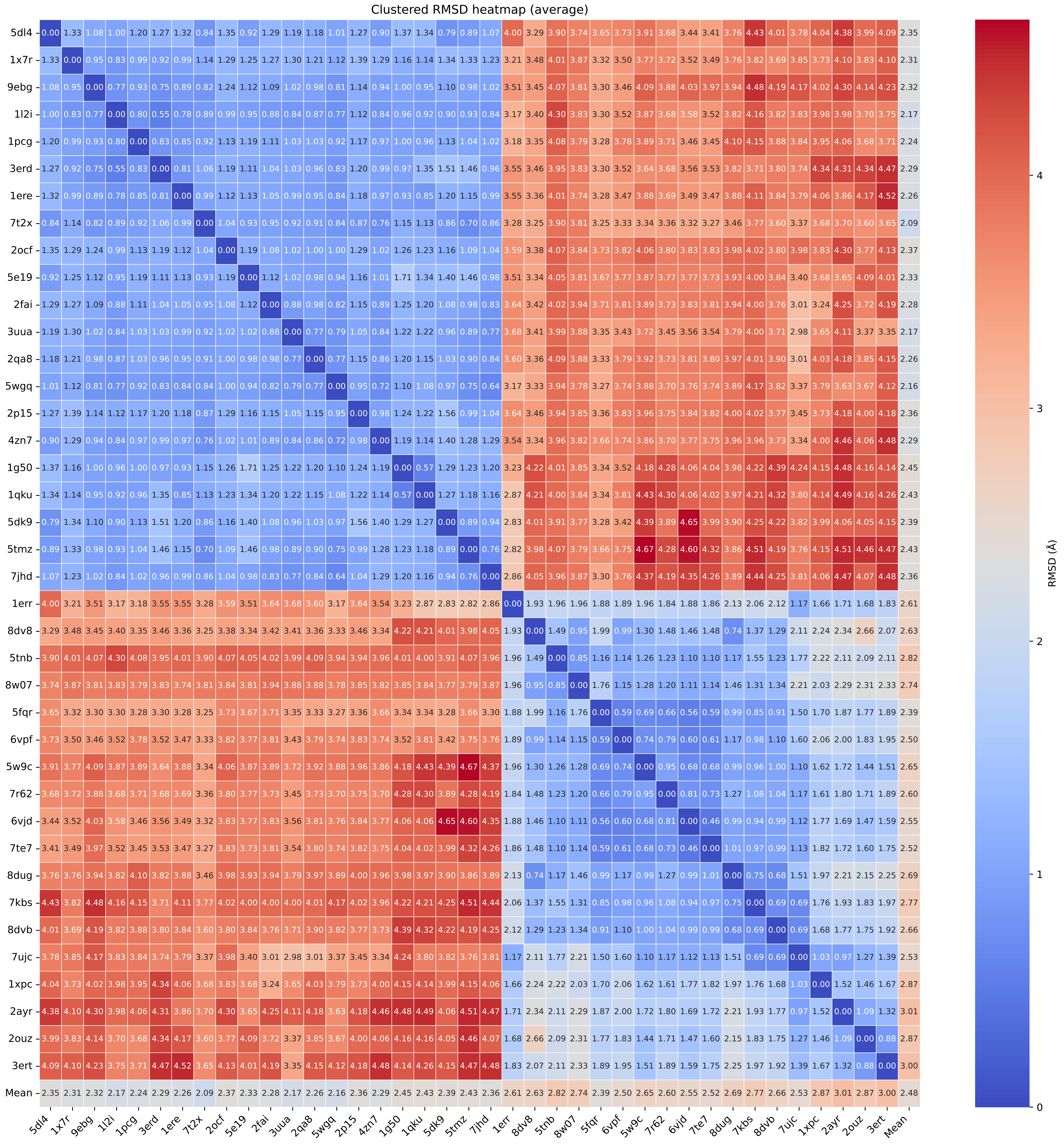

Clustered RMSD heatmap (average)
5dl4
1x7r
9ebg
1l2i
1pcg
3erd
1ere
7t2x
2ocf
5e19
2fai
3uua
2qa8
5wgq
2p15
4zn7
1g50
1qku
5dk9
5tmz
7jhd
1err
8dv8
5tnb
8w07
5fqr
6vpf
5w9c
7r62
6vjd
7te7
8dug
7kbs
8dvb
7ujc
1xpc
2ayr
2ouz
3ert
Mean
RMSD (Å)
0
1
2
3
4

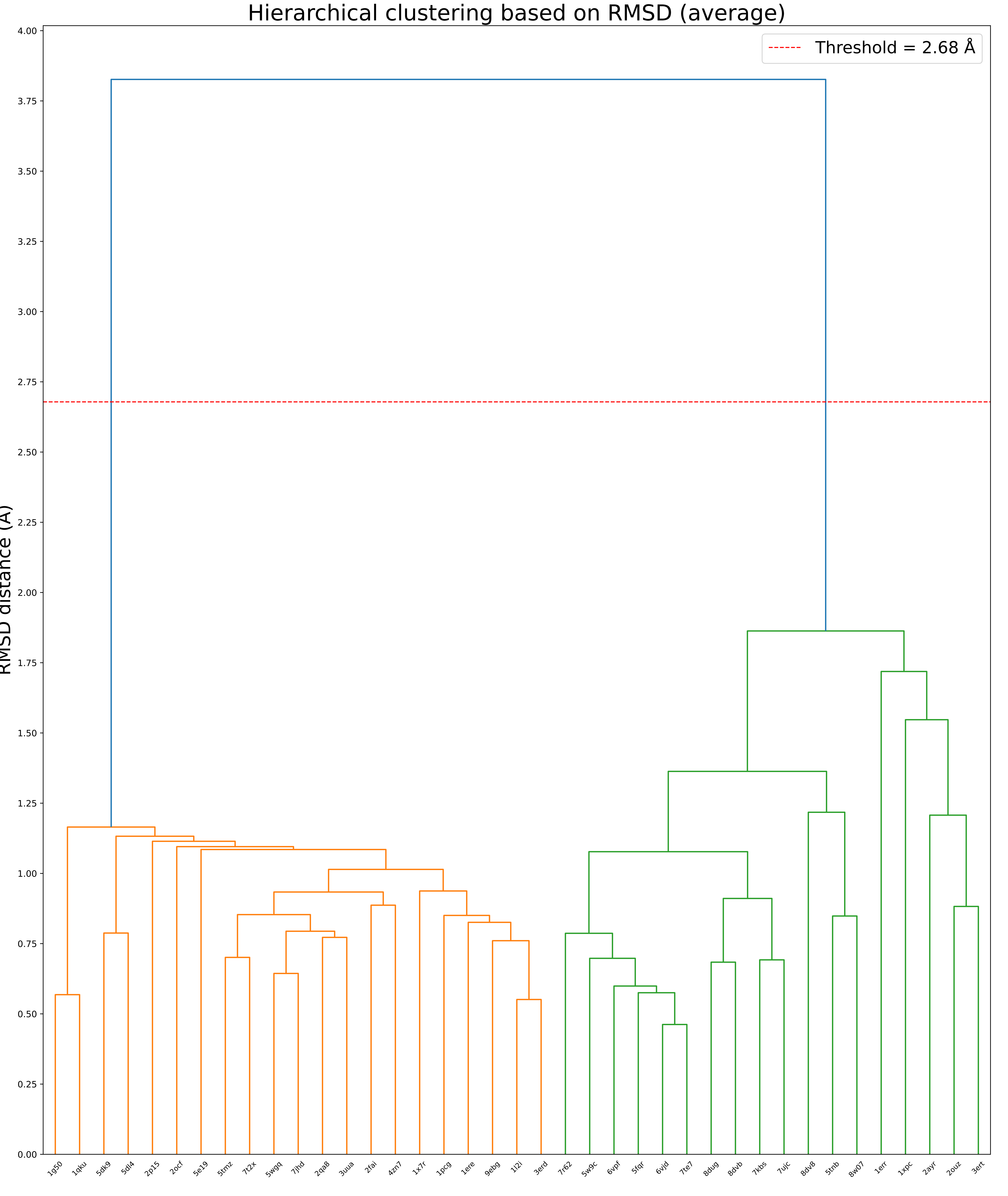

Hierarchical clustering based on RMSD (average)
Threshold = 2.68 Å
RMSD distance (Å)
4.00
3.75
3.50
3.25
3.00
2.75
2.50
2.25
2.00
1.75
1.50
1.25
1.00
0.75
0.50
0.25
0.00
1g50 1qku 5dk9 5dl4 2p15 2ocf 5e19 5tmz 7t2x 5wgq 7jhd 2qa8 3uua 2fai 4zn7 1x7r 1pcg 1ere 9ebg 1l2i 3erd 7r62 5w9c 6vpf 5fqr 6vjd 7te7 8dug 8dvb 7kbs 7ujc 8dv8 5tnb 8w07 1err 1xpc 2ayr 2ouz 3ert
